# Intra-arterial infusion of bevacizumab for the treatment of peritoneal metastatic high-grade renal cell carcinoma: a case report

**DOI:** 10.3389/fimmu.2026.1686650

**Published:** 2026-03-09

**Authors:** Song Jin, Chaoming Dai, Xiaomeng Dai, Peng Zhao, Bin Zhou, Jizhou Zhang, Weijia Fang

**Affiliations:** 1 Department of Oncology, Wenzhou Traditional Chinese Medicine Hospital of Zhejiang Chinese Medical University, Wenzhou, Zhejiang, China; 2 Department of Medical Oncology, The First Affiliated Hospital, School of Medicine, Zhejiang University, Hangzhou, China; 3 Department of Infectious Diseases, Wenzhou Traditional Chinese Medicine Hospital of Zhejiang Chinese Medical University, Wenzhou, Zhejiang, China

**Keywords:** arterial embolism, bevacizumab, local treatment, metastatic renal cell carcinoma, peritoneal metastasis

## Abstract

**Background:**

Metastatic renal cell carcinoma (mRCC) is a common type of urologic malignancy with limited systemic treatment, especially for extensive metastatic lesions in the peritoneum and greater omentum.

**Case report:**

A 35-year-old male patient was admitted to the hospital with left lumbar pain, leading to the discovery of left renal mass. He subsequently underwent a nephrectomy, with postoperative pathology confirming RCC. Following surgery, he received three cycles of interleukin-2 therapy. Within 5 months of nephrectomy, related examinations confirmed tumor recurrence and metastasis, and palliative surgery was performed. Postoperative pathology confirmed high-grade RCC, diagnosed as stage IV with pancreatic, adrenal, and splenic metastases. The patient then underwent first-line treatment with ICI (Immune Checkpoint Inhibitors) in combination with pezopanib for 9 months before progressing and then changing to second-line treatment with ICI in combination with axitinib for 2 months before progressing again. The patient was subsequently screened for clinical studies and rapidly progressed to extensive abdominal metastases after 1 month. The patient was then treated with bevacizumab abdominal artery perfusion embolization and achieved partial remission. Subsequent systemic therapy was changed to everolimus combined with voronib targeted therapy until now, and the lesion has continued to shrink on multiple follow-ups in between.

**Conclusion:**

Abdominal arterial infusion of bevacizumab embolization provides a feasible local treatment option for peritoneal metastases in stage IV RCC, warranting further clinical exploration.

## Introduction

Renal cell carcinoma (RCC) represents one of cancer with high incidence among malignant tumors of the urinary system, accounting for approximately 2%-3% of all adult cancers (1). Patients diagnosed with stage IV RCC frequently present with multiple organ metastases, which present significant therapeutic challenges and are associated with a poor prognosis (2).

Currently, the treatment of advanced RCC is predominantly relied on targeted therapy and immunotherapy. However, in patients particularly with relapsed peritoneal metastases, the efficacy of systemic therapies is often limited (3). In recent years, localized therapeutic approaches such as tumor resection, stereotactic body radiotherapy, and radiofrequency ablation have shown promise in managing RCC which might be able to meet the unmet need in clinical practice (4–6).

In this case, the patient with stage IV RCC underwent super-selective arterial embolization using bevacizumab combined with sequential back-line targeted therapy to address abdominal metastases, demonstrating remarkable therapeutic efficacy and offering a promising therapeutic strategy for similar clinical scenarios.

## Case report

A 35-year-old male presented with lumbar pain to an external hospital in November 2022. Imaging studies identified a renal mass, leading to a radical nephrectomy. Pathological examination of the surgical specimen confirmed a left renal cell carcinoma (RCC) measuring 15.0 cm x 5.0 cm, with areas of poor differentiation. The ureteral margin was negative. Postoperatively, the patient received three courses of interleukin-2 (IL-2) therapy.

In April 2023, a PET-CT scan demonstrated tumor recurrence involving the pancreatic tail and spleen. Subsequently, on May 4, 2023, the patient underwent distal pancreatectomy, total splenectomy, unilateral adrenalectomy, and diaphragmatic repair. Postoperative pathology confirmed high-grade RCC, supported by immunohistochemistry (IHC) results: CK (pan) (+), PAX-8 (+), Vimentin (+), Ki-67 (60% +), GATA-3 (focal weak +), FH (+); CK7 (–), CD10 (–), CD117 (–), TFE3 (–), CAIX (–), TTF-1 (–), PTEN (–), HMB45 (–). Postoperative pathological staging:pTxN0M1.IMDC stratification: intermediate risk.

The patient received first-line therapy with toripalimab (ICI, 240 mg, every 3 weeks) combined with pazopanib (400 mg, twice daily) from July 11, 2023, to April 22, 2024. After 8 months of treatment, tumor recurrence was observed, manifesting as peritoneal metastases. CT imaging revealed nodular thickening of the left anterior abdominal wall and haziness of the greater omentum, consistent with disease progression (PD). Second-line treatment, initiated on May 6, 2024, involved toripalimab (240 mg, every 3 weeks) combined with axitinib (5 mg, twice daily). After 2 months of treatment, contrast-enhanced CT demonstrated PD. The patient was then screened for clinical trial, and one month later, imaging reexamination found extensive peritoneal metastasis and significant enlargement of lesions, suggesting rapid progression. On August 28, 2024, superselective arterial angiography with intra-arterial infusion of bevacizumab followed by embolization was performed. Iodixanol angiography revealed that the hepatic lesion was supplied by the left hepatic artery, while the peritoneal lesions were supplied by two branches of the omental artery. A total of 500 mg of bevacizumab was administered, with the dose calculated at 7.5 mg/kg. This dosage was adopted based on a Phase II, multicenter clinical trial that evaluated the efficacy and safety of intra-arterial bevacizumab in patients with colorectal cancer-related hepatic metastases (7). was infused into the left hepatic artery and the two omental artery branches, followed by embolization using gelatin sponge particles. On October 9, 2024, the patient was subsequently switched to everolimus (5 mg, once daily) combined with vorolanib (200 mg, once daily) as part of subsequent-line targeted therapy.

**Figure 1:**
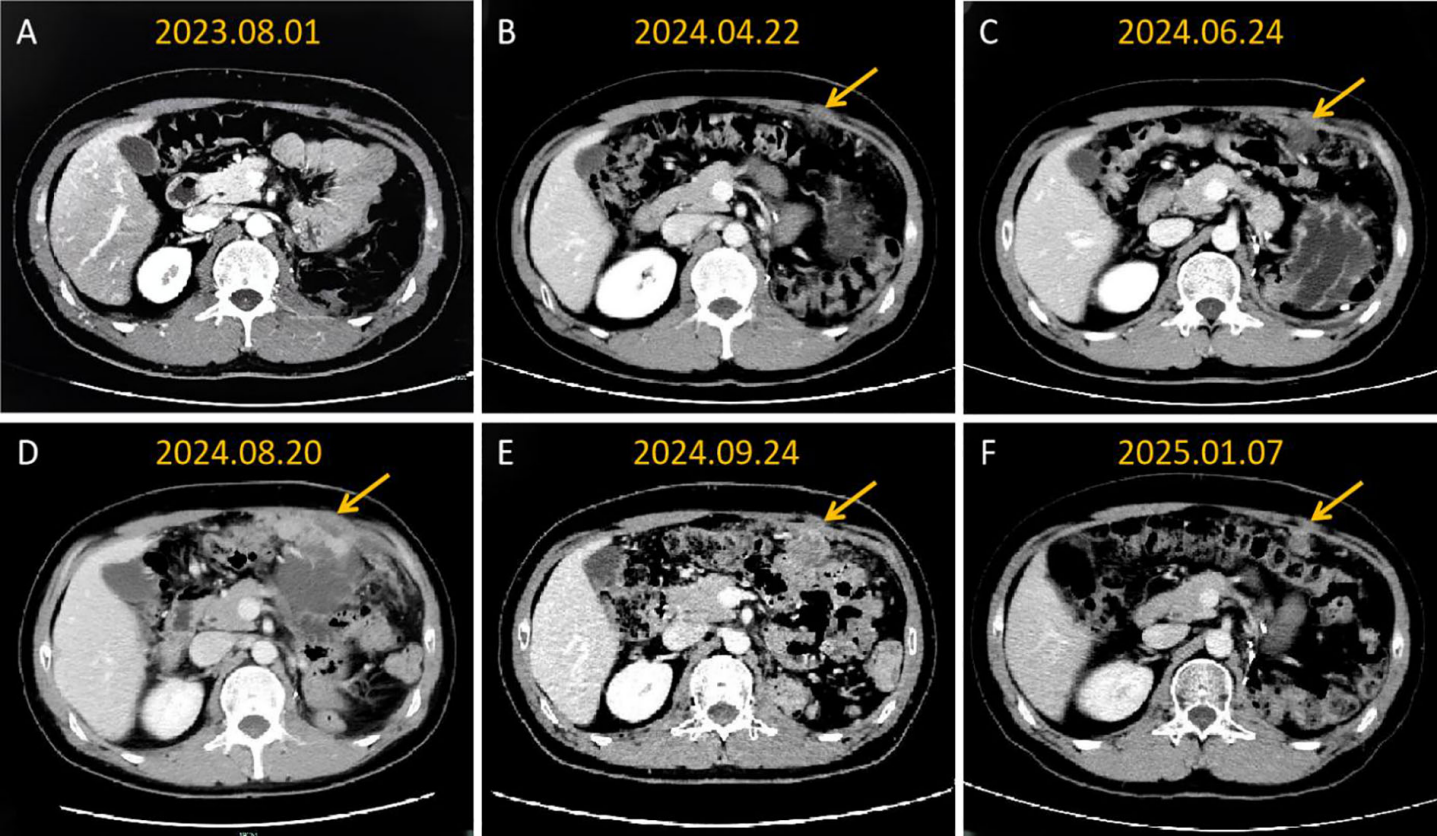
Contrast-enhanced CT imaging follow-up of abdominal wall metastatic lesions confirms sustained partial remission with sequential targeted therapy with abdominal artery infusion of bevacizumab. **(A)** The abdominal cavity shows postoperative changes, with no metastatic lesions observed; **(B)** Local nodular thickening begins to appear on the left anterior abdominal wall, suggesting recurrence and metastasis; **(C)** The metastatic tumor on the left anterior abdominal wall has increased in size; **(D)** The abdominal wall lesions have significantly enlarged, and extensive diffuse implantation metastasis occurs in the abdominal cavity; **(E)** After one month of local treatment, the abdominal wall and greater omentum lesions have significantly shrunk; **(F)** The abdominal cavity lesions continue to shrink.

**Figure 2:**
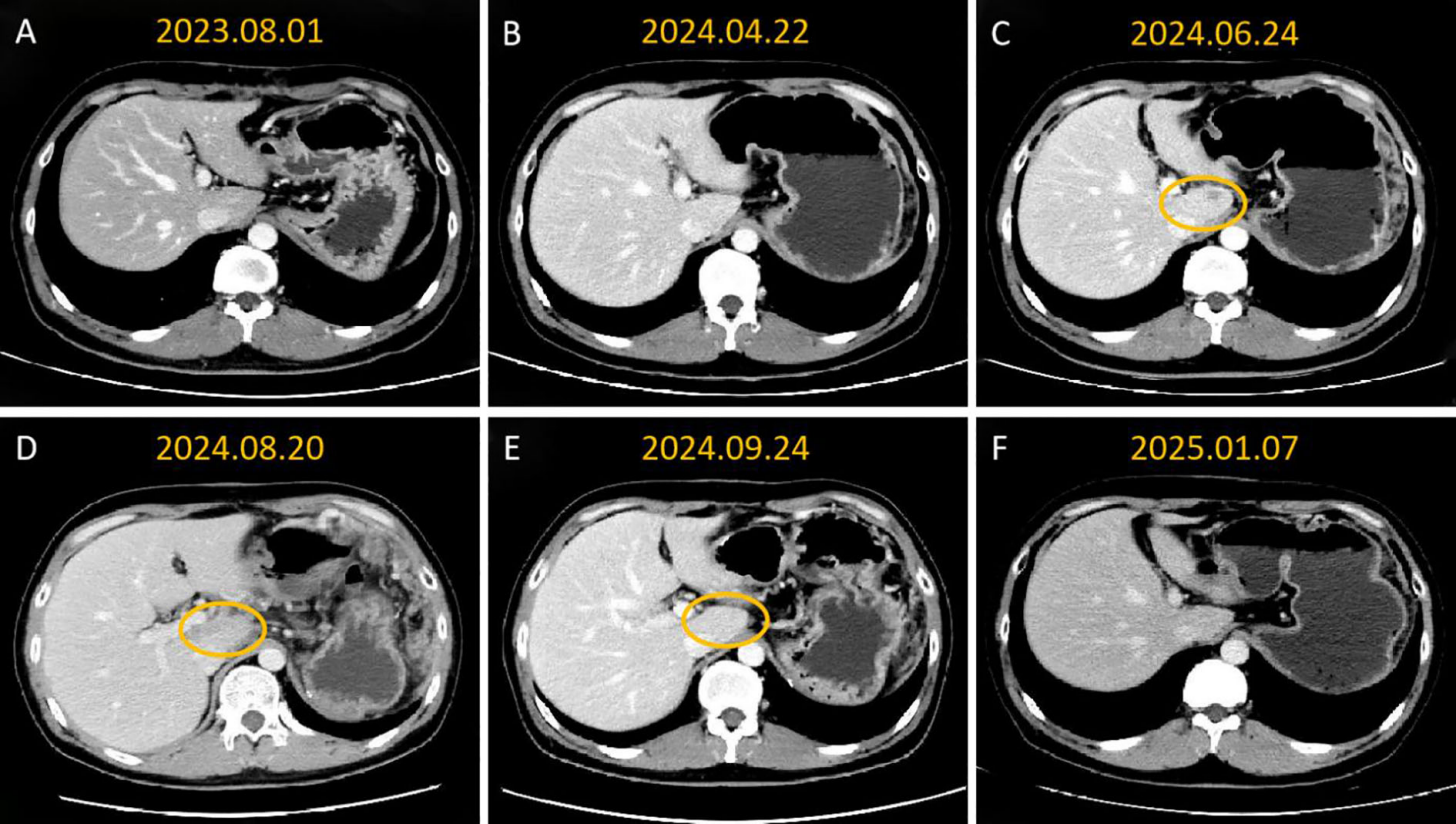
Contrast-enhanced CT imaging showed that sequential targeted therapy with intraperitoneal artery infusion of bevacizumab resulted in sustained partial remission of hepatic caudal metastasies. **(A, B)** no lesions were found in the liver during the re-examination; **(C)** metastatic tumors began to appear in the caudate lobe of the liver; **(D)** the metastatic tumors in the caudate lobe of the liver increased in number and size; **(E)** after one month of local treatment, the liver lesions significantly shrank and decreased; **(F)** The liver lesions had receded and these lesions were not visualized clearly.

**Figure 3:**
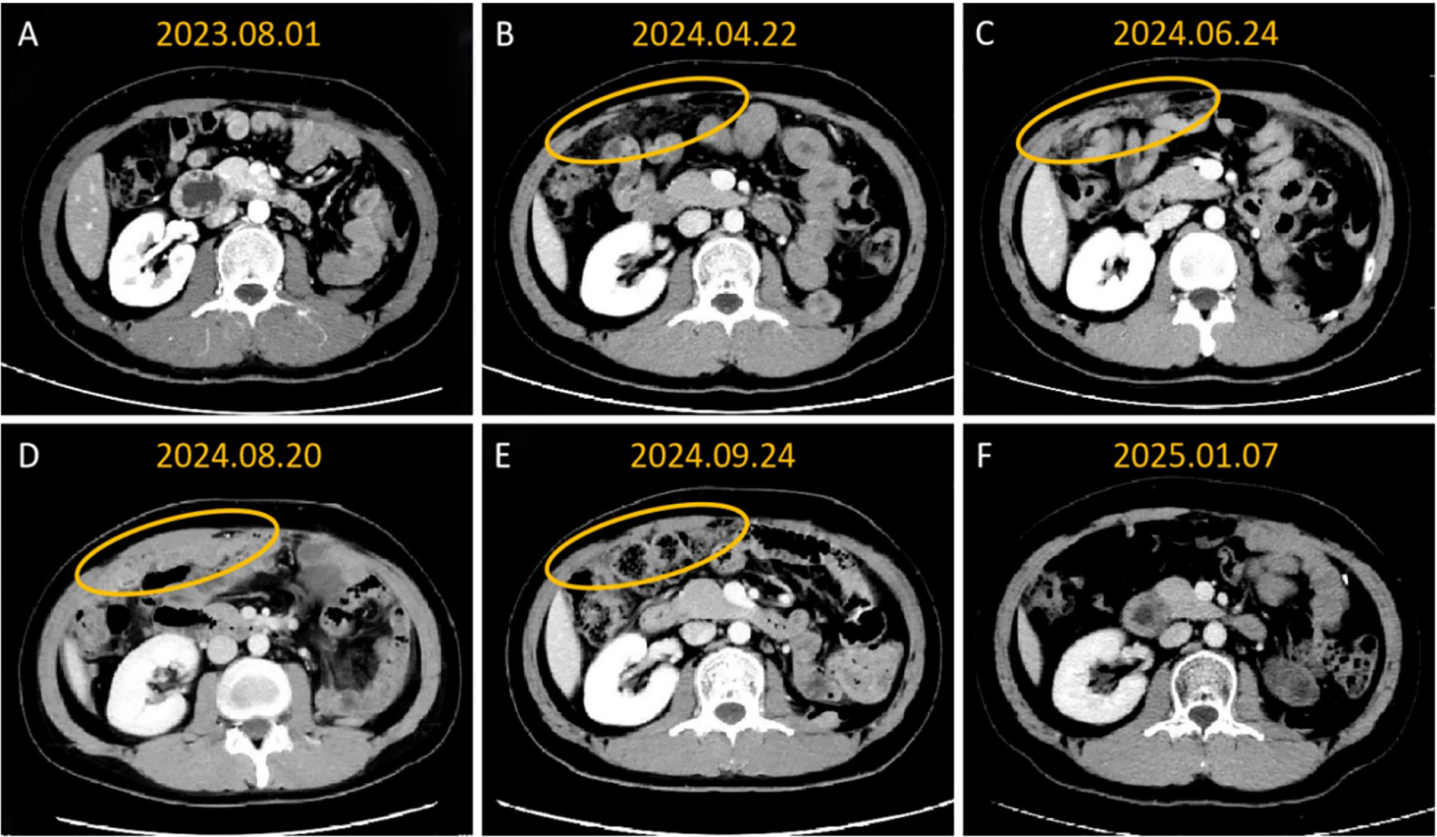
Contrast-enhanced CT imaging showed that sequential targeted therapy with intraperitoneal artery infusion of bevacizumab resulted in sustained partial remission of greater omental metastasies. **(A)** The abdominal cavity shows postoperative changes; **(B)** The greater omentum begins to show thickening and turbidity, suggesting recurrence and metastasis; **(C)** The metastatic tumor in the greater omentum has enlarged; **(D)** The lesion in the greater omentum is significantly thickened in a pancake-like manner; **(E)** The lesion in the greater omentum has significantly shrunk; **(F)** The greater omentum lesion has receded and is not clearly visible.

Follow-up results showed significant improvement. On September 24, 2024, follow-up CT demonstrated evidence of substantial regression of left hepatic and abdominal metastatic lesions, with an objective partial response (PR) documented (**Figure 1**), and the reduction of all target lesions exceeded 50%. By January 2025, follow-up contrast-enhanced CT revealed complete resolution of hepatic lesions and further reduction in abdominal metastatic lesions (**Figures 2**, **3**). No adverse reactions related to bevacizumab were observed, and the treatment was well-tolerated.

## Discussion

Patients with stage IV high-grade RCC generally have a poor prognosis, with a 5-year survival rate of approximately 10%. In this case, the patient achieved significant disease control through super-selective arterial infusion of bevacizumab combined with embolization as a local treatment. Subsequent sequential targeted therapy resulted in continued shrinkage of the lesions during the rapid progression of the disease. The patient achieved a progression-free survival (PFS) of 6 months.

RCC is notable as a highly angiogenic primary malignancy, with elevated microvessel density in tumor tissues correlating with a worse prognosis (8). Pathologically, QIAN et al. classified RCC vasculature based on CD31 and CD34 expression into two categories: undifferentiated vessels (CD31+/CD34-) and differentiated vessels (CD31+/CD34+) (9). Undifferentiated vessels are characterized by the absence or minimal presence of lumens, thicker vessel walls, and smaller volumes compared to differentiated vessels. Higher densities of undifferentiated vessels are associated with shorter survival, higher tumor grade, faster progression of the primary tumor, and resistance to anti-angiogenic therapies. Anti-angiogenic therapies are widely used in clinical practice for treating RCC. However, resistance and disease progression following systemic anti-angiogenic small-molecule tyrosine kinase inhibitor therapies, such as pazopanib and axitinib, remain significant clinical challenges. Bevacizumab, as an anti-angiogenic agent, works by inhibiting tumor growth and metastasis through blockade of the VEGF signaling pathway (10).

The case demonstrates that two months after ICI combined with targeted therapy, patients developed rapid progression of intra-abdominal metastases. During this phase, bevacizumab infusion therapy was administered via intraperitoneal perfusion. While the observed clinical and imaging improvements could potentially be attributed to the combination of toripalimab and axicabtagene ciloleucel, the primary driver of these improvements was identified as bevacizumab. Intra-arterial infusion of bevacizumab via the celiac artery has demonstrated effectiveness in controlling peritoneal metastatic lesions for metastatic RCC in this patient. The therapeutic efficacy of this approach can be attributed to several key mechanisms. Super-elective intra-arterial infusion and embolization allow for the direct delivery of bevacizumab to peritoneal metastatic sites, significantly increasing local drug concentration while minimizing systemic side effects. This targeted delivery enhances the inhibitory effect on local tumor tissue and induces tumor vascular ischemia. Furthermore, local embolization blocks the tumor blood supply, accelerating ischemic necrosis of the tumor tissue (11). Additionally, the high local concentrations of bevacizumab may modulate the immune microenvironment by inhibiting vascular endothelial growth factor (VEGF). This alteration enhances anti-tumor immune responses, further contributing to the therapeutic effects (12). Approximately one month after bevacizumab abdominal artery perfusion embolization treatment, a follow-up examination was conducted, revealing a PR, which suggested the efficacy of bevacizumab artery perfusion embolization. Subsequently, a continuous oral regimen of everolimus and voronidine was initiated. Further follow-up examinations showed sustained PR, highly suggesting the therapeutic effects of everolimus and voronidine. However, it must be noted that the possibility of sustained or synergistic effects of bevacizumab cannot be entirely ruled out. Moreover, locally infused bevacizumab can potentially “normalize” the tumor vasculature, improving the hypoxic tumor microenvironment. This normalization enhances the efficacy of subsequent systemic therapies, such as the combination of vorolanib and everolimus (13). These mechanisms collectively highlight the potential of intra-arterial bevacizumab infusion as a novel treatment strategy for metastatic RCC.

In conclusion, this case highlights that super-selective intra-arterial infusion of bevacizumab presents a novel and effective local treatment option for patients with stage IV high-grade RCC, particularly those with relapsed peritoneal metastases. Future validation through larger-scale clinical studies is necessary to establish the broader applicability and safety of this therapeutic strategy.

## Data Availability

The datasets presented in this study can be found in online repositories. The names of the repository/repositories and accession number(s) can be found in the article/supplementary material.

## References

[1] Hsieh JJ Purdue MP Signoretti S Swanton C Albiges L Schmidinger M . Renal cell carcinoma. Nat Rev Dis Primers. (2017) 3:17009. doi: 10.1038/nrdp.2017.9, PMID: 28276433 PMC5936048

[2] Ficarra V Righetti R Pilloni S D’Amico A Maffei N Novella G . Prognostic factors in patients with renal cell carcinoma: retrospective analysis of 675 cases. Eur Urol. (2002) 41:190–8. doi: 10.1016/S0302-2838(01)00027-6, PMID: 12074408

[3] Barragan-Carrillo R Saad E Saliby RM Sun M Albiges L Bex A . First and second-line treatments in metastatic renal cell carcinoma. Eur Urol. (2025) 87:143–54. doi: 10.1016/j.eururo.2024.10.019, PMID: 39505582

[4] Lam JS Shvarts O Pantuck AJ . Changing concepts in the surgical management of renal cell carcinoma. Eur Urol. (2004) 45:692–705. doi: 10.1016/j.eururo.2004.02.002, PMID: 15149740

[5] Meyer E Pasquier D Bernadou G Calais G Maroun P Bossi A . Stereotactic radiation therapy in the strategy of treatment of metastatic renal cell carcinoma: A study of the Getug group. Eur J Cancer. (2018) 98:38–47. doi: 10.1016/j.ejca.2018.04.008, PMID: 29864737

[6] Ali M Mooi J Lawrentschuk N McKay RR Hannan R Lo SS . The role of stereotactic ablative body radiotherapy in renal cell carcinoma. Eur Urol. (2022) 82:613–22. doi: 10.1016/j.eururo.2022.06.017, PMID: 35843777

[7] Rigault E Lacas B Glehen O Smith D Dupont-Bierre E Guimbaud R . Intra-arterial hepatic bevacizumab and systemic chemotherapy in hepatic metastasis of colorectal cancer: A phase II multicentric trial in second-line treatment. Cancer Treat Res Commun. (2023) 34:100674. doi: 10.1016/j.ctarc.2022.100674, PMID: 36565566

[8] Jilaveanu LB Puligandla M Weiss SA Wang XV Zito C Flaherty KT . Tumor microvessel density as a prognostic marker in high-risk renal cell carcinoma patients treated on ECOG-ACRIN E2805. Clin Cancer Res. (2018) 24:217–23. doi: 10.1158/1078-0432.CCR-17-1555, PMID: 29066509 PMC5904512

[9] Yao X Qian CN Zhang ZF Tan MH Kort EJ Yang XJ . Two distinct types of blood vessels in clear cell renal cell carcinoma have contrasting prognostic implications. Clin Cancer Res. (2007) 13:161–9. doi: 10.1158/1078-0432.CCR-06-0774, PMID: 17200351

[10] Rini BI Small EJ . Biology and clinical development of vascular endothelial growth factor-targeted therapy in renal cell carcinoma. J Clin Oncol. (2005) 23:1028–43. doi: 10.1200/JCO.2005.01.186, PMID: 15534359

[11] D’Amico RS Khatri D Reichman N Patel NV Wong T Fralin SR . Super selective intra-arterial cerebral infusion of modern chemotherapeutics after blood-brain barrier disruption: where are we now, and where we are going. J Neurooncol. (2020) 147:261–78. doi: 10.1007/s11060-020-03435-6, PMID: 32076934

[12] Fukumura D Kloepper J Amoozgar Z Duda DG Jain RK . Enhancing cancer immunotherapy using antiangiogenics: opportunities and challenges. Nat Rev Clin Oncol. (2018) 15:325–40. doi: 10.1038/nrclinonc.2018.29, PMID: 29508855 PMC5921900

[13] Cao Y Langer R Ferrara N . Targeting angiogenesis in oncology, ophthalmology and beyond. Nat Rev Drug Discov. (2023) 22:476–95. doi: 10.1038/s41573-023-00671-z, PMID: 37041221

